# The Therapeutics of Venesection

**Published:** 1882-06

**Authors:** 


### The Therapeutics of Venesection.

Dr. Wm. A. Dunn, in a paper read at the meeting of the section for Clinical Medicine and Pathology of the Suffolk District Medical Society, summarizes his views as follows:

1. That although the errors of former days, without doubt, allowed a very great abuse of venesection, it has sufficient merit as a therapeutic agent to demand our earnest consideration.

2. If we are sincere in following the motto of our Society, *Natura duce*, we shall take the suggestions which nature gives and bleed in carefully selected cases.

3. That in febrile attacks a loss of blood will lower the temperature, and this decrease in temperature is known to be disproportionate to the amount of blood lost.

4. That by venesection we do not actually diminish the volume of blood, but we cause the blood to become more watery, the free passage of the blood through the pulmonary circuit seems to be promoted, and the functional labor which the lungs have to perform is diminished by the abstraction of a certain number of the more solid particles.

5. It is fallacious to depend upon the condition of the pulse alone as the criterion of the amount of blood to be removed, or the benefit which the patient derives by a venesection. After a venesection the pulse sometimes appears to indicate increased power of the heart's action. The artery seems to strike against the finger with more force than before the abstraction of blood. Formerly practitioners were misled by this effect upon the pulse, and blood-letting was employed as a means of increasing the power of the heart's action. The sensation which the finger receives is delusive, and is caused by the quickness of the movements of the artery. This has been shown by the sphygmograph to depend on the diminished tension of the arteries following the abstraction of blood. It is to be borne in mind, says Flint, in estimating the power of the heart's action by the sensible characters of the pulse, that the sense of resistence which is felt and the amount of pressure required to impress the artery are the evidence of strength.—*Boston Med. and Surg. Jour.*

